# Exploiting macrophages as targeted carrier to guide nanoparticles into glioma

**DOI:** 10.18632/oncotarget.9464

**Published:** 2016-05-18

**Authors:** Liang Pang, Jing Qin, Limei Han, Wenjie Zhao, Jianming Liang, Zhongyi Xie, Pei Yang, Jianxin Wang

**Affiliations:** ^1^ Key Laboratory of Smart Drug Delivery, Ministry of Education, Department of Pharmaceutics, School of Pharmacy, Fudan University, Shanghai, 201203, China; ^2^ Shanghai Institute of Pharmaceutical Industry, Shanghai, 201203, China

**Keywords:** macrophages, glioma, inflammation, hypoxic, nanoparticles

## Abstract

The restriction of anti-cancer drugs entry to tumor sites in the brain is a major impediment to the development of new strategies for the treatment of glioma. Based on the finding that macrophages possess an intrinsic homing property enabling them to migrate to tumor sites across the endothelial barriers in response to the excretion of cytokines/chemokines in the diseased tissues, we exploited macrophages as ‘Trojan horses’ to carry drug-loading nanoparticles (NPs), pass through barriers, and offload them into brain tumor sites. Anticancer drugs were encapsulated in nanoparticles to avoid their damage to the cells. Drug loading NPs was then incubated with RAW264.7 cells *in vitro* to prepare macrophage-NPs (M-NPs). The release of NPs from M-NPs was very slow in medium of DMEM and 10% FBS and significantly accelerated when LPS and IFN-γ were added to mimic tumor inflammation microenvironment. The viability of macrophages was not affected when the concentration of doxorubicin lower than 25 μg/ml. The improvement of cellular uptake and penetration into the core of glioma spheroids of M-NPs compared with NPs was verified in *in vitro* studies. The tumor-targeting efficiency of NPs was also significantly enhanced after loading into macrophages in nude mice bearing intracranial U87 glioma. Our results provided great potential of macrophages as an active biocarrier to deliver anticancer drugs to the tumor sites in the brain and improve therapeutic effects of glioma.

## INTRODUCTION

The treatment of glioma is one of the greatest challenges in cancer therapy [1]. Despite the substantial progress of current treatment strategies in recent decades, the prolongation of glioma patients’ survival has not been efficiently achieved [2]. Since infiltrative growth of glioma leads to incomplete surgical excision [3], radiotherapy and chemotherapy are necessary following surgery. Therefore, how to deliver drugs into tumor site represents one of the most important obstacles during the treatment of glioma.

In the early stage of glioma, an endothelial cell monolayer associated with pericytes and astrocytes constitutes the blood-brain barrier (BBB), which protects brain tissue from harmful substances in blood circulation [4–6]. Meanwhile, it prevents therapeutic drugs from entering the brain to treat various diseases. With the progression of glioma into later stage, the integrity of BBB is compromised due to enhanced permeability and retention (EPR) effect [7]. However, the increased interstitial fluid pressure (IFP) inside the tumor and the blood tumor barrier (BTB) still impede therapeutic agents into tumor [8, 9]. Only by overcoming these barriers, the drug could be successfully delivered into the diseased site. Recently, multifunctional nano-drug delivery systems have been developed to improve therapeutic effect of different drugs [10], but rapid clearance from blood, limited targeting to diseased tissues and serious immunogenicity seriously restricted their application in tumor therapy.

To our knowledge, when inflammation happens, leukocyte will be mobilized from bone marrow into circulation and move into inflammatory site. Some studies have exploited this pathological property to design cell-based drug delivery system. For instance, monocytes were used as drug carrier to treat atherosclerosis with high efficiency [11]. As with other inflammatory responses, inflammation in the brain is also characterized by extensive leukocytes infiltration into brain tissue by cell diapedesis and chemotaxis [12–14]. Brynskikh et al. utilized macrophage as a drug vehicle to improve the delivery of redox enzymes into the brain for neuroprotection of dopaminergic neurons in a mouse model of Parkinson's disease. Therapeutic efficacy of macrophages loaded with nanozyme was confirmed by twofold reductions in microgliosis and twofold increase in tyrosine hydroxylase-expressing dopaminergic neurons [15].

Rudolf Virchow identified the presence of leukocytes within tumors for the first time in the 19th century, which indicated a possible link between inflammation and cancer [16]. Inflammation is a critical component in the progression of tumor, including initiation, promotion, invasion and metastasis [16, 17]. A larger number of immune cells, mainly macrophages and T cells, are recruited into tumor microenvironments. It has been reported that macrophages constitute up to a third of the whole tumor mass in glioma [18]. Additionally, hypoxia is the hallmark feature of most solid tumors due to their rapid growth and poorly organized vasculature. Such hypoxic pressure impedes the penetration of anticancer drugs into tumor tissues. Therefore, the hypoxic regions in tumor are usually resistant to radio-and chemotherapy [19, 20]. Interestingly, the chemoattractant released by tumor cells in response to hypoxia attract macrophages infiltration into tumor tissues [21, 22]. Huang et al. employed bone marrow-derived monocytes to deliver polymer bubbles and vesicles for chemotherapy of tumor hypoxia [23]. Inspired by these understandings, a novel strategy utilizing macrophage as a carrier to migrate across the BBB, BBTB and home into tumor sites is conceived. Importantly, macrophages are able to carry drugs into brain tumor throughout the whole progress.

**Figure 1:**
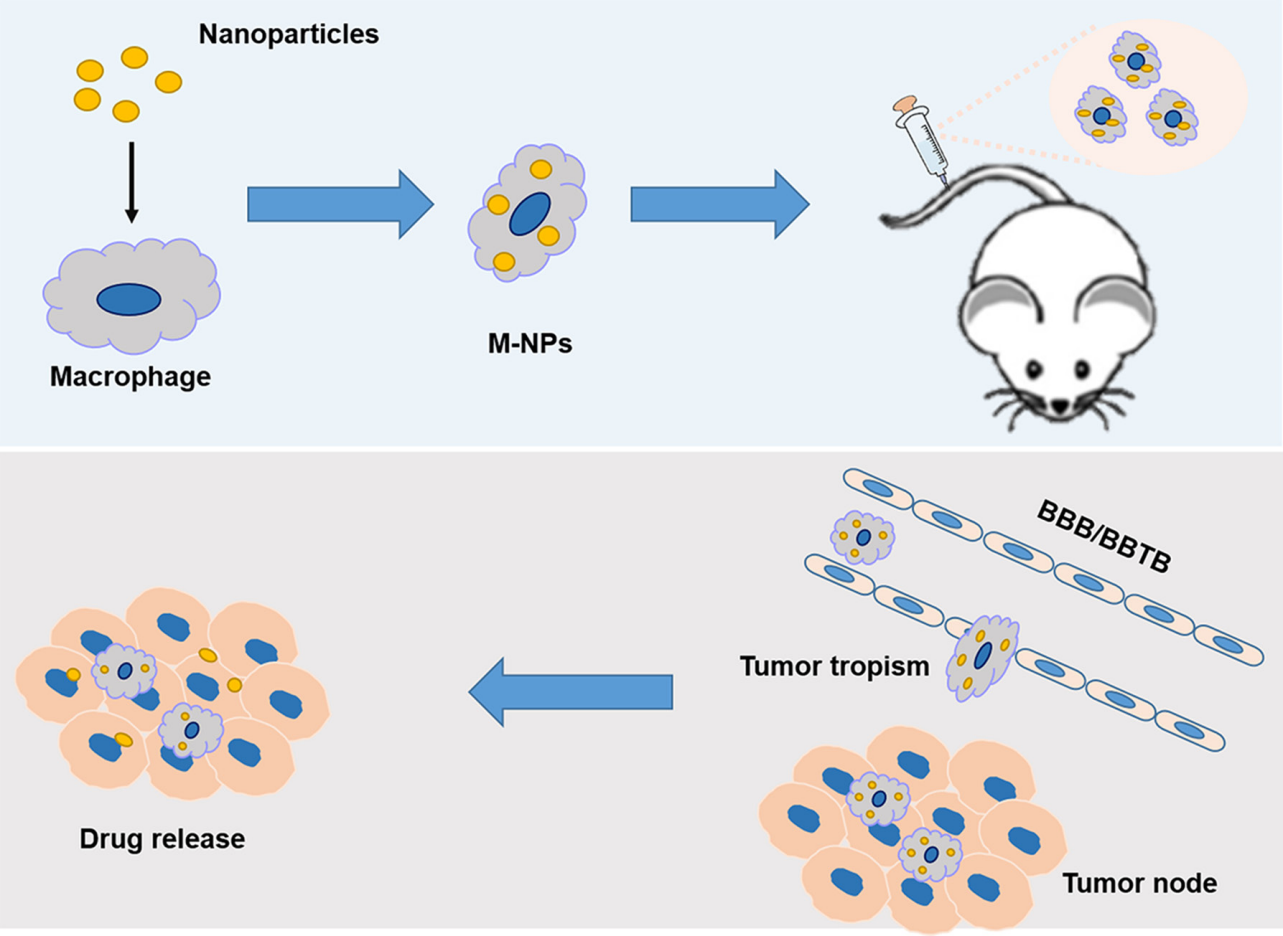
Schematic illustration of the construction of ‘Macrophage-NPs’ and their targeting delivery into brain tumor

In this manuscript, RAW264.7, a kind of mouse macrophage-like cell line with similar functions to primary macrophage cells, were used here to demonstrate the feasibility of macrophage as vehicle to deliver drug into glioma. Figure 1 illustrates schematic strategy adopted in this work for the construction of M-NPs and *in vivo* fate. A fluorescent dye, coumarin-6, and a near infrared dye, DiR, were respectively encapsulated to quantitatively or qualitatively track the behavior of a macrophage based drug delivery system. The stability of this system and its release kinetics in a simulated inflammatory environment was studied. Avascular U87 glioma spheroids were employed to explore the penetration ability of M-NPs system. The tumor targeting capacity of this system was validated in orthotopic U87 glioma bearing mice model by *in vivo* imaging system, and the brain distribution was evaluated by confocal microscopy in frozen brain slices.

## RESULTS

### Characterization of nanoparticles

In order to study the effect of particle size on the uptake efficiency of macrophages, nanoparticles in three sizes (50–100 nm, 100–200 nm, 200–300 nm) were prepared by emulsion-solvent evaporation method. The particle size, Zeta potential and polydispersity index (PDI) of the nanoparticles were listed in Table 1. Encapsulation of coumarin-6, DiR did not significantly influence the characteristics of nanoparticles. Owing to the water solubility of doxorubicin hydrochloride, the DOX-NPs were prepared by double-emulsion method. The size, zeta potential and PDI of DOX-NPs is 141.6 nm, −31.7 mv and 0.086, respectively.

**Table 1:** Characterization of nanoparticles

Nanoparticles	Mean Size (nm)	Polydispersity (PDI)	Zeta potential (mV)
NP-Small	69.90 ± 3.730	0.29 ± 0.034	− 41.33 ± 3.092
NP-Middle	138.13 ± 2.205	0.10 ± 0.007	− 42.93 ± 1.305
NP-Large	236.67 ± 9.730	0.20 ± 0.029	− 43.90 ± 0.436

### Effect of particle size on macrophages uptake

**Figure 2:**
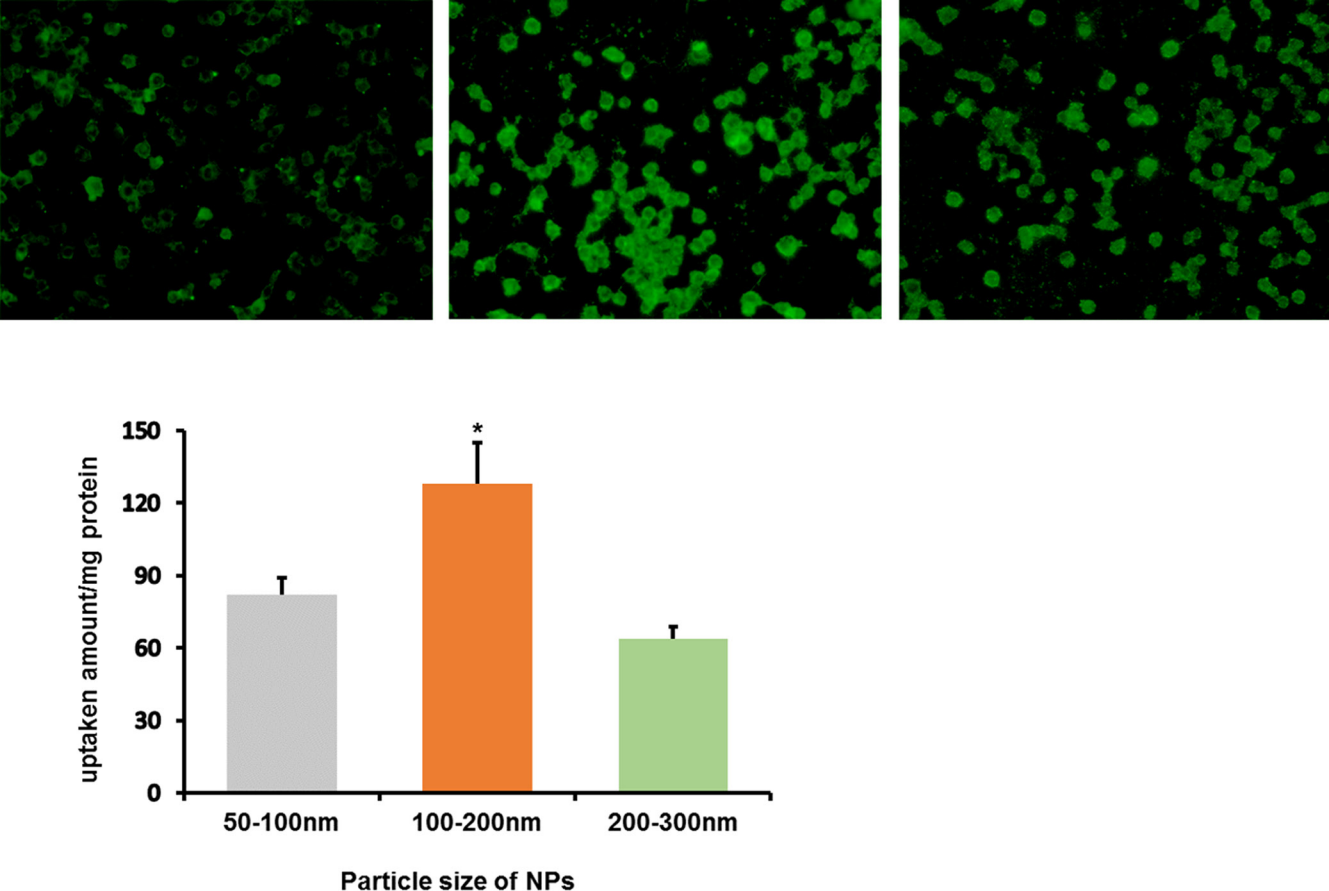
*In vitro* cellular uptake of coumarin-6-labeled NPs in three sizes by RAW264.7 after incubation for 2 h (**A**) Left: 50–100 nm; Middle: 100–200 nm; Right: 200–300 nm. (**B**) The quantitative results of cellular uptake for RAW264.7, *P < 0.05, compared with other two groups.

Macrophages itself could efficiently phagocytize nanoparticles by endocytosis. The size of NPs influences the phagocytosis capacity of macrophages greatly. Coumarin-6 was used as fluorescent probe to investigate the cellular uptake characteristics. As illustrated in Figure 2, qualitative fluorescent images showed that macrophages incubated with 100–200 nm NPs exhibited the highest fluorescence intensity among three types of NPs under the same incubation conditions. Quantitatively, the cellular uptake of 100–200 nm NPs was1.56 and 2.0 fold of the uptake efficiency of 50–100 nm and 200–300 nm NPs, respectively.

### Effect of DOX-NPs loading on macrophages viability

**Figure 3:**
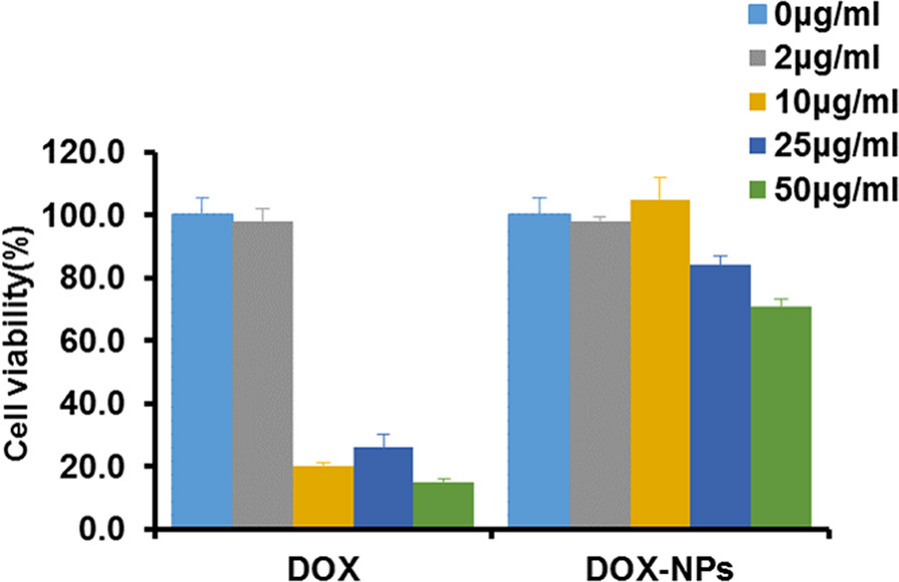
Macrophages viability after incubation with DOX or DOX-NPs for 12 h at 37°C

The function of macrophage as carrier is strongly correlated with its viability after the loading of DOX-NPs. Macrophages showed reduced viability after incubation with 10, 25, and 50 μg/ml free DOX. Whereas, incubation with DOX-NPs resulted in higher viability than that of free DOX under the same concentration (Figure 3). Hence, in a certain concentration range of DOX, DOX-NPs was successfully loaded with low toxicity into macrophages, leading us to conclude that macrophages would be a useful candidate as a biocarrier to deliver nanodrugs. It is worthwhile to note that the incubation concentration of NPs and cell viability should be carefully balanced because high drug concentration may cause toxicity to macrophages.

### Release profile of NPs from macrophages

**Figure 4:**
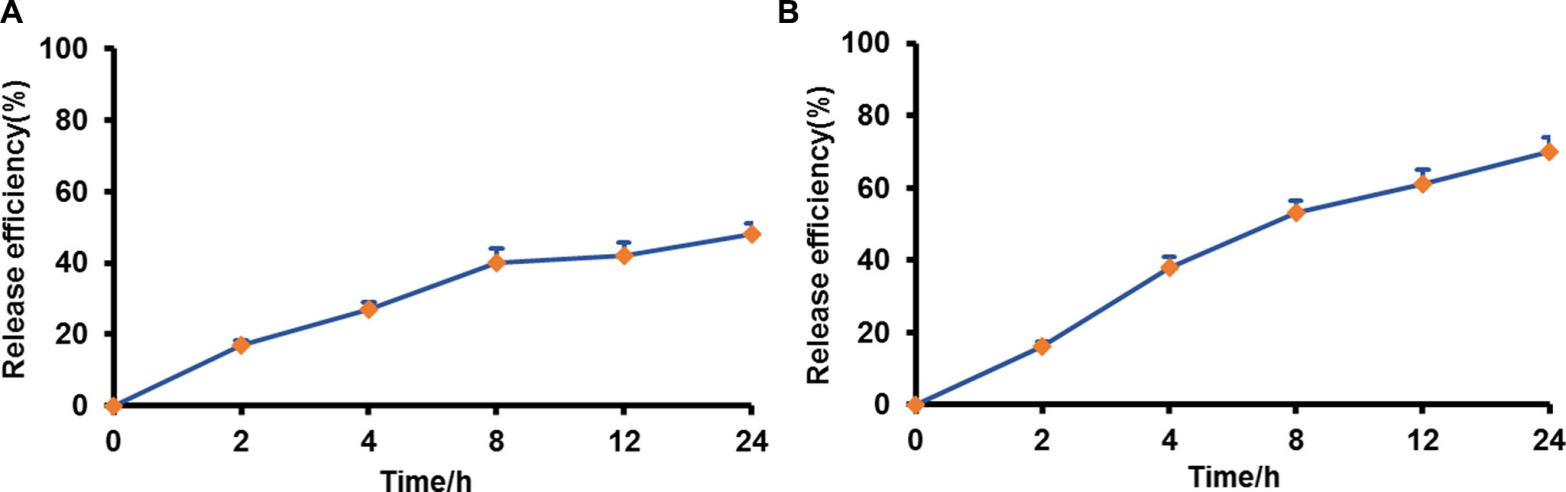
Drug release from pre-loaded macrophages in different medium (**A**) DMEM, 10% FBS; (**B**) DMEM, 10% FBS, LPS and IFN-γ.

DiR was used to track the release profile of NPs from macrophages. The cells were pre-loaded with nanoparticles for 2 hours, then washed with phosphate buffered saline (PBS) and incubated in fresh media for different time intervals (0 h, 2 h, 4 h, 8 h, 12 h, 24 h). The media was collected and the fluorescence intensity was measured by fluorospectro photometer. Sustained release of DiR-NPs from macrophages was observed and achieved cumulative release of 42% after 24 h incubation in Dulbecco's Modified Eagle Medium (DMEM) containing 10% fetal bovine serum (FBS). Meanwhile, a faster release pattern was obtained (71%) in DMEM containing 10% FBS with addition of LPS and IFN-γ (Figure 4), which indicated that drug release would be accelerated in tumor microenvironment.

### *In vitro* glioma spheroid penetration of M-NPs

**Figure 5:**
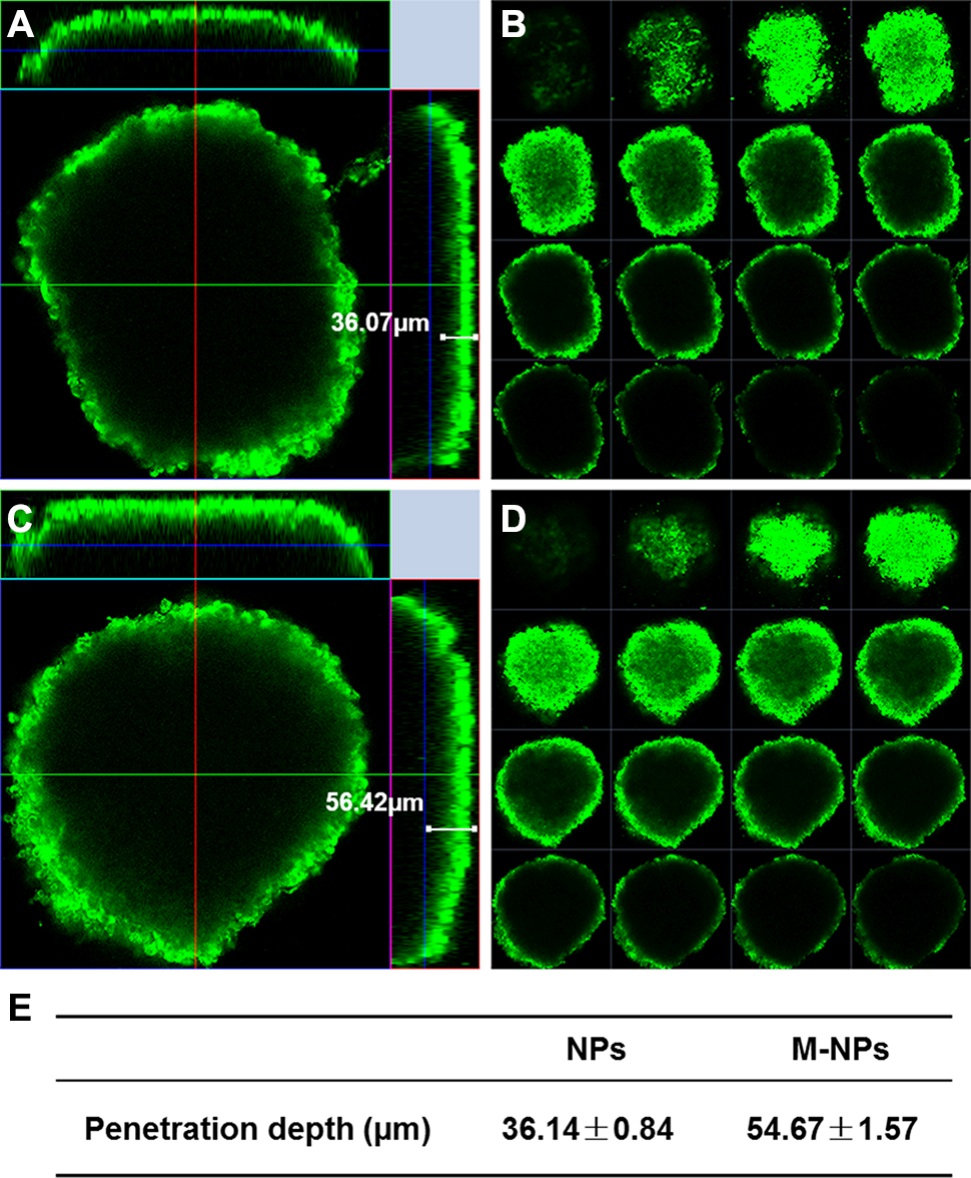
Penetration of coumarin-6-labeled NPs (A, B) and M-NPs (C, D) into U87 glioma spheroids after incubation for 12 h (A and C) penetration depth of NPs and M-NPs; (B and D) multi-level scan of the penetration of NPs and M-NPs with intervals of 20 μm; (E) the value of penetration depth were expressed as mean ± standard deviation (*n* = 3), *P* < 0.05.

*In vitro* U87 glioma spheroids model was established to evaluate the penetration ability of M-NPs. After 12 h incubation, M-NPs showed more extensive infiltration into tumor spheroids than NPs. M-NPs could reach about 56.42 μm away from the rim of the spheroids, and was 1.56 fold deeper than that of NPs which penetrated only 36.07 μm into glioma spheroids (Figure 5A, 5C). Multi-level scanning from the top of the glioma spheroid with an interval 20 μm into the core showed that the fluorescence intensity of M-NPs treatment is higher than that of NPs (Figure 5B, 5D). Therefore, nanoparticles loaded in macrophage could not only facilitate the uptake by tumor cells, but also enhance their penetration into tumor spheroids.

### Tumor targeting of M-NPs

**Figure 6:**
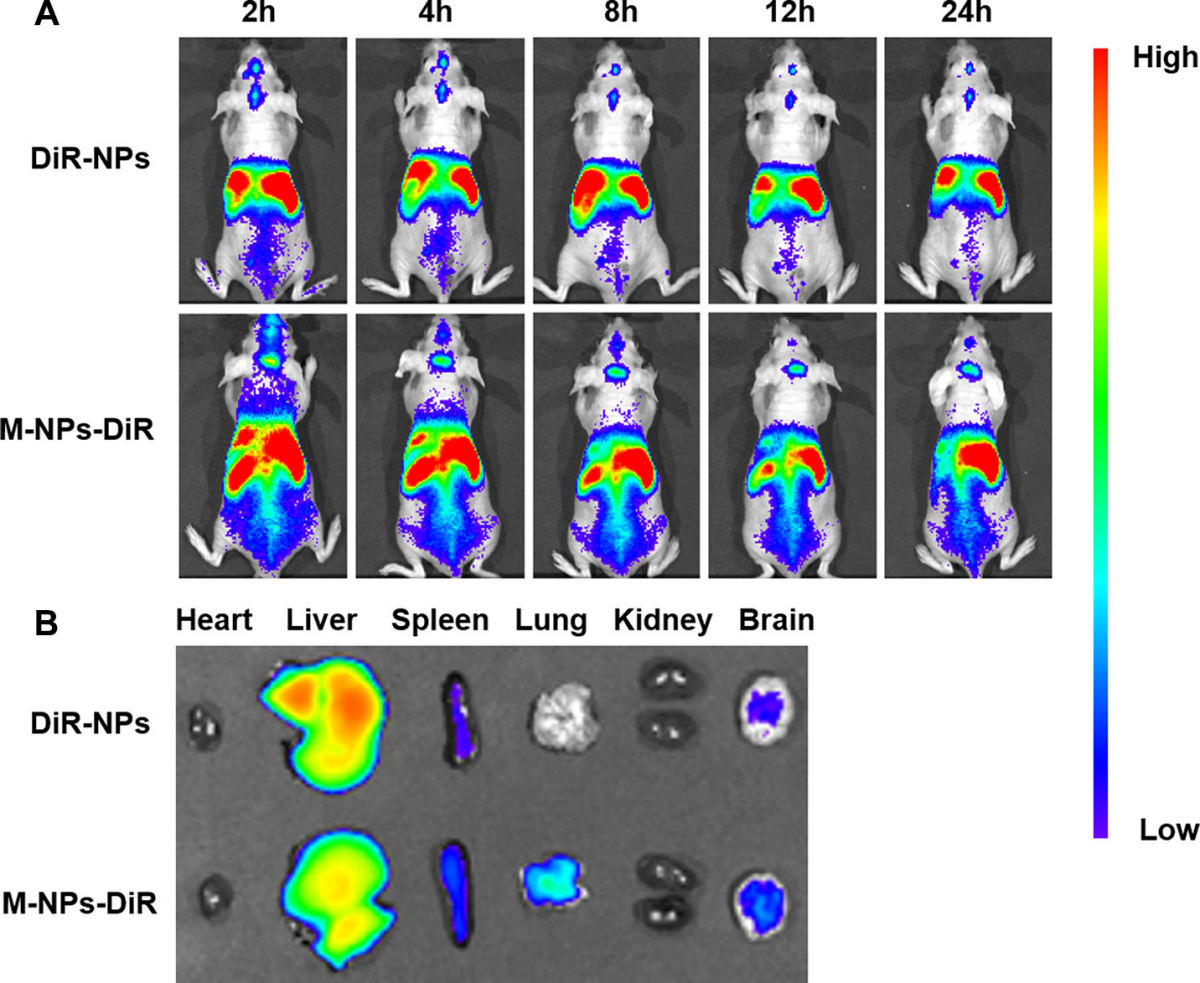
*In vivo* imaging of brain glioma-bearing nude mice administrated with DiR-labeled NPs and M-NPs at different time points (A), *Ex vivo* imaging of major organs collected at 24 h after dosing (B)

To determine the biodistribution of NPs and M-NPs, *in vivo* imaging was conducted to track the particles in nude mice bearing intracranial U87 glioma. Both the NPs and M-NPs could apparently accumulate in the tumor tissues from 0.5 h after injection (data not shown). However, the fluorescence intensity of M-NPs treated mice was much higher than that of NPs treated mice at all-time points from 2 to 24 h (Figure 6), indicating that macrophage as cell carrier significantly improved the tumor-targeting efficiency of NPs. Correspondingly, the conclusion was further confirmed by *ex vivo* imaging of the brains.

### *In vivo* tumor localization of M-NPs

**Figure 7:**
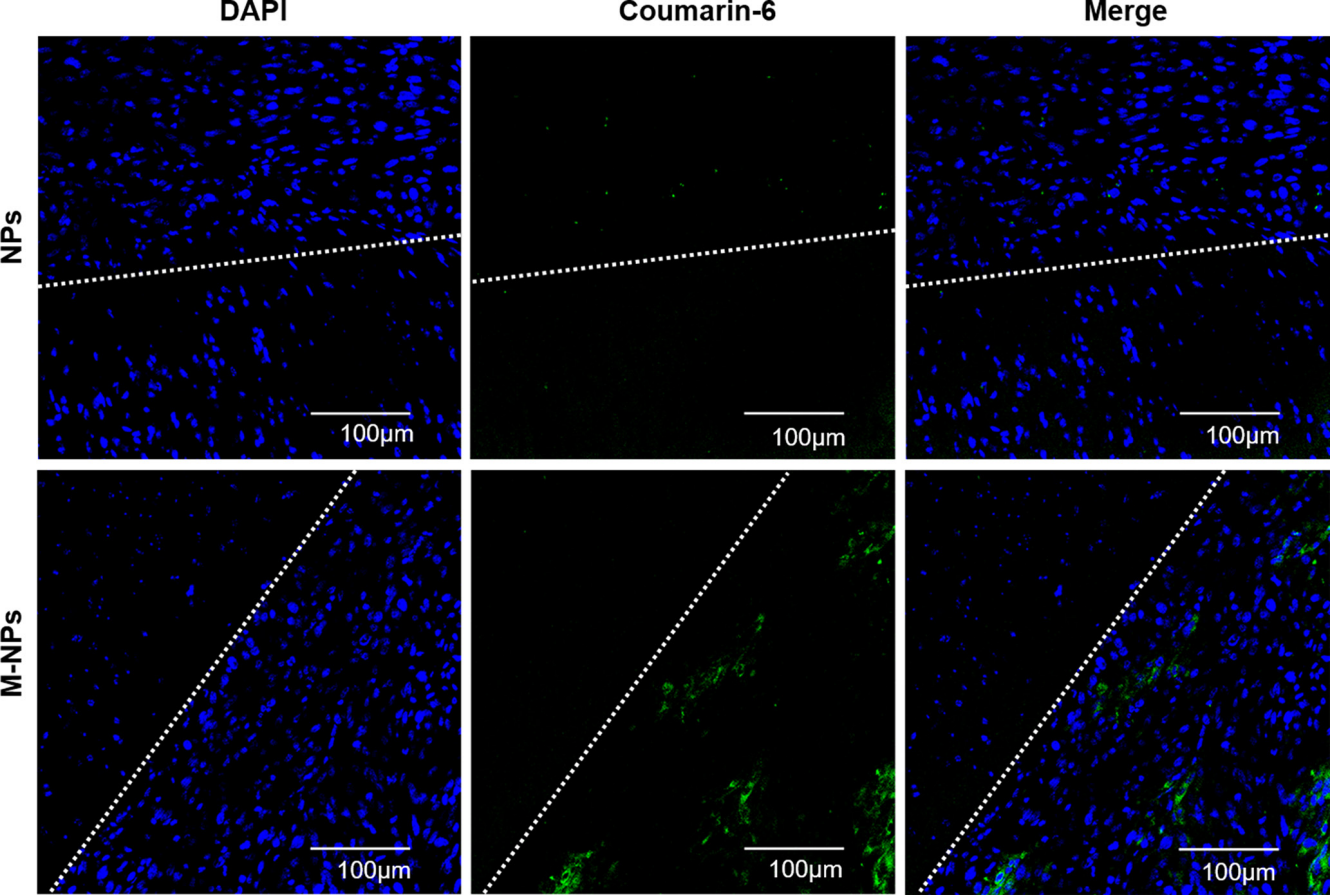
*In vivo* glioma distribution of coumarin-6-labeled NPs and M-NPs 12 h after administration Blue: DAPI stained cell nuclei, Green: Coumarin-6-labeled NPs, White line: border of the glioma, Dense area: glioma tissue, Sparse area: brain tissue.

Three weeks after glioma cell inoculation, *in vivo* brain distribution of coumarin 6-labeled NPs and M-NPs was measured 12 h after intravenous administration into mice. As shown in Figure 7, there was only a little green fluorescence distributed in glioma tissues in NPs group. but in the case of the M-NPs group, an obvious stronger fluorescent signal was detected and a much deeper permeation was observed at the glioma parenchyma. The results indicated that macrophage as carrier can increase the accumulation of NPs in brain tumor.

## DISCUSSION

During the progression of glioma, tumor tissues are protected by BBB, BBTB and high IFP, which make the parenchyma inaccessible to therapeutic drugs [4, 7, 8]. Nanoparticle drug delivery systems with active targeting capabilities have been explored for enhancing drug delivery to glioma by conjugating target moiety onto the surface of nanoparticles [24, 25]. In recent decades, cell based drug delivery systems take advantage of circulatory cell (red blood cell, T cell, macrophage, antigen presenting cell, etc.) to improve the therapeutic effect of anti-cancer drugs [15, 26, 27]. Using cells as carriers for drug delivery offers several advantages over free drug, including improved drug efficacy, extended half-lives, sustained drug release, and limited immunogenicity and cytotoxicity.

When tumor occurs, the tumor inflammation environment could induce the overexpression of the cell adhesion moleculars (CAMs) on the surface of endothelial cell monolayer, which mediate interaction between macrophages and endothelial cells, facilitate the initial process of macrophage rolling, firm attachment to endothelium and transmigration [28, 29]. Meanwhile, there are accumulating evidences showing that a larger number of macrophages are attracted and retained in hypoxia regions by local synthesis of chemoattractant in tumor cells undergoing hypoxia due to rapid tumor growth [20, 21]. We compared the chemotactic ability of unactivated RAW264.7 and activated RAW264.7 by boyden chamber method. The migration rate of unactivated and activated RAW264.7 is 9.28 ± 0.54% and 11.06 ± 0.53%, respectively. No significant difference was found between them. Therefore, unactivated RAW264.7 cell line was chosen in this manuscript.

If free anticancer drugs are encapsulated into cells, it may cause damage to the carrier itself before arriving at tumor sites and suppress the functions of cells as transporter. Therefore, we encapsulated anticancer drugs into nanoparticles with the purpose to reduce the damage of the drug to the cell carriers [30]. As illustrated in Figure 2, CCK8 assay showed that the viability of macrophages incubated with NPs-DOX was higher than those incubated with free DOX. By blocking direct contact between the cell and the drug, DOX-NPs efficiently reduced drug-induced cellular toxicity. The properties of nanoparticles, such as size, shape, chemical functionality and surface charge, are closely related to the uptake capacity of macrophages [31]. Nanoparticles in three sizes were prepared by the same method using the same materials. Under identical conditions, 100–200 nm nanoparticles are easily internalized by macrophages compared with other two particles of different size, and the loading of NPs did not affect the migration of macrophages into tumor tissues [32].

Experiments performed on 3D glioma spheroids investigated the migratory potential of macrophages loaded with nanoparticles. Tumor cells in spheroids display higher resistance to radio- and chemotherapy than monolayer tumor cells, and are thought to mimic tumor nodes well prior to vascularization *in vivo* [33, 34]. As illustrated in Figure 5, NPs loaded macrophages infiltration toward spheroids was observed to be 1.5-fold deeper penetration into the spheroids than free NPs. These results demonstrated that macrophage as an anticancer agent transporter could enhance drug delivery in inaccessible tumor hypoxic region effectively.

*In vivo* imaging experiments were performed to evaluate the behavior of M-NPs in nude mice bearing intracranial U87 glioma. The accumulation of NPs in tumors via passive EPR effect was limited, while M-NPs exhibited a significant superiority in glioma targeting with high fluorescent intensity at all-time points. Consistently, in frozen brain sections, the accumulation of NPs was low and located on the border of glioma. However, M-NPs showed an extensive distribution and deep penetration into glioma parenchyma, indicating circulating macrophages could overcome the barriers (BBB, BBTB, IFP) and penetrate into the tumor tissue. The major organs, including heart, liver, spleen, lung, kidney, and brain, were harvested 24 hours after the administration of M-NPs and imaged under *ex vivo* fluorescence. The results illustrated in Figure 6B showed that there were some differences in tissues distribution between NPs and M-NPs, especially in the lung, which might due to pulmonary capillaries retention of macrophages.

It was reported that therapeutically meaningful amount of free DOX could be loaded into the RAW264.7 cells by short time incubation, About 65% of the drug were released from the cells in the first 2 h [35]. Less than 20% and only 42% NPs were released from preloaded macrophages after 2 h and 24 h incubation respectively, indicating the sustained release of NPs from macrophages. The release of drug from macrophages is a complicated process. Based on the results of our study and literature [13, 35, 36], we speculated that the drug could be released from macrophages in two ways. Firstly, NPs was excreted from macrophages by exocytosis, and the free drug diffused from NPs into extracellular medium subsequently. Secondly, the free drug might be released from NPs within cells, and then diffuse into the surroundings via passive driving force caused by concentration gradient between cells and surroundings, or the multi-drug resistant proteins P-gp expressed in the macrophage could pump the drug out of the cells. The former plays a dominant role in the process. Once NPs loading macrophages enter into the tumor sites, tumor inflammation environment will activate macrophages and result in significant increase in drug release from macrophages. It was found that when the cell carrier enters into diseased site in Parkinson's disease, the direct contact between cell carrier and endothelial, neuronal and glial cells promote drug transfer through endocytosis-independent mechanism, which mainly involve fusion of cell membranes, bridging conduits and nanoparticle lipid coating [37]. Therefore, it is supposed that the increased nanoparticles transfer might be occurred in a similar way after macrophage being attracted into tumor tissue. The inner of tumor tissue is filled with inflammatory cytokines [16, 17]. In order to mimic the inflammatory microenvironment in tumor tissue, LPS and IFN-γ were added to the medium according to previous reports [42, 43]. When LPS and IFN-γ were added, they would bind with Toll-like receptor 4 and IFN-γ receptor expressed on macrophage respectively, activate RAW264.7 and finally promote drug release by exocytosis.

The study demonstrated the feasibility of using macrophages as carriers for targeting anticancer drug into glioma. Considering that the M2 phenotype macrophage in tumor promotes tumor growth and contributes to tumor angiogenesis, its migration into tumor would weaken the effect of anticancer agents [38], the M1 type macrophage maybe the best candidate as a cell carrier since it resist tumor progression. Our ultimate goal is to encapsulate nanodrugs into patient derived M1 type macrophage, then transfer the macrophage-NPs back into the patient to achieve improved efficacy and to reduce immune responses.

## MATERIALS AND METHODS

### Reagents

PLGA (LA: GA = 75:25, Mw: 12,000 Da) was kindly provided by Evonik (Germany). Emprove exp poly (vinyl alcohol) (PVA) 4–88 was given as a present from Merck (Darmstadt, Germany). DiR (1, 1′-dioctadecyl-3, 3, 3′, 3′-tetramethyl indotricarbocyanine Iodide), Coumarin-6 was purchased from Caliper (USA), Aladdin (Shanghai, China) respectively. DAPI (4, 6-diamidino-2-phenylindole) was purchased from Beyotime (Haimen, China). Doxorubicin hydrochloride (DOX·HCL) was obtained from Melonapharma (Dalian, China). Lipopolysaccharide (LPS) from Sigma (St. Louis, MO, USA) and IFN-γ from Peprotech (Rokey Hill, USA) were used. All cell culture regents were purchased from Corning, Inc. (VA, USA) except Gibco fetal bovine serum.

### Cell culture

RAW264.7 cell lines, obtained from the Chinese Academy of Sciences Cells Bank (Shanghai, China), were cultured in Dulbecco's Modified Eagle Medium (DMEM) supplemented with 10% FBS, 1% L-glutamine, 1% antibiotics and 1% nonessential amino acids at 37°C, 5% CO_2_, and 95% humidity in a CO_2_ incubator.

### Animals

Balb/c nude mice (Female, 4–5 weeks, 20–22 g) were obtained from the Shanghai B&K Lab Animal Ltd. (Shanghai, China) and housed under standard conditions with free access to food and water. The protocol of animal study was approved by the Animal Experimentation Ethics Committee of Fudan University.

### Preparation of NPs

PLGA nanoparticles in three sizes loaded with fluorescent dye were prepared by emulsion/solvent evaporation method according to the procedure reported previously [39]. The particle size could be controlled by adjusting PLGA amount, emulsifier concentration, ultrasonic time. Briefly, 5 mg, 20 mg, 150 mg PLGA and 100 μl coumarin 6 (1 mg/ml) or 10 μl DiR (5 mg/ml) were dissolved in 1 ml dichloromethane respectively, to which 2 ml of different concentration (0.05%, 0.5%, 2.0% respectively) of sodium cholate aqueous solution was added, with the mixture sonicated on ice using a probe sonicator (Scientz Biotechnology Co., Ltd., China). Then the emulsion was dispersed into 18 ml of corresponding concentration of sodium cholate aqueous solution under rapid magnetic stirring for 60 min. After evaporating dichloromethane with a ZX-98 rotary evaporator (LOOYE, China) at 40°C, the suspensions were centrifuged using a TJ-25 centrifuge (Beckman Counter, USA). After discarding the supernatant, the obtained nanoparticles were re-suspended with 0. 1 M PBS buffer (pH 7.4) and stored at 4°C for further use.

DOX·HCL loaded nanoparticles were prepared by the double/emulsion method as previously reported [40, 41]. 20 mg PLGA was dissolved in 1 ml of ethyl acetate followed by addition of 100 μl of DOX·HCL (10 mg/ml), the first emulsion was formed by tip sonication in ice bath, to which 2 ml of 2% PVA was added immediately followed by sonication in ice bath to finally form double emulsion. The emulsion was dispersed into 9 ml of 2%PVA with magnetic stirring in room temperature (600 rpm) for 2 hours. The organic solvent was removed by vacuum evaporation at 40°C for 20 min. The residues were concentrated by centrifugation using a TJ-25 centrifuge.

The physiochemical parameters of NPs, including particle size, zeta potential, polydispersity were measured using a dynamic light scattering detector (Zetasizer, Nano-ZS, Malvern, UK).

### Preload of NPs in macrophages

RAW264.7 were seeded into 12-well plates at a density of 2 × 10^4^ cells/ml. 24 h later, the cells were incubated with 200 ng/mL coumarin-6-loaded NPs in three particle sizes (50–100 nm, 100–200 nm, 200–300 nm) respectively in the absence of FBS for 2 h (*n* = 3). After being rinsed with PBS three times, the cells were harvested and probe sonicated in ice bath, then centrifuged at 8000 rpm for 10 min. The supernatant is collected. One half was measured for protein concentration, the other for fluorescence intensity. The fluorescence intensity was normalized for protein content and expressed in fluorescence intensity per mg of protein. Meanwhile, the pre-loaded cells were washed three times with PBS and observed under fluorescent microscopy (Leica, DMI4000D, Germany) immediately.

### Cell viability assay

5 × 10^3^ RAW264.7 in 100 μl medium were cultured into each well of a 96-well plate. Free DOX·HCL or DOX-NPs were then added at concentrations 2, 10, 25 and 50 μg/ml. Macrophages were incubated with DOX or DOX-NPs for 12 h. At the end of incubation, the culture medium was discarded and the cells were washed with PBS. Cell counting kit-8 (CCK-8) (Beyotime, Nantong, China) was used to test viability of macrophages by incubating with 100 μl of fresh medium containing 10 μl of CCK8 solution for 3 h at 37°C in 5% CO2. The absorbance of medium was measured at 450 nm using a multimode reader (Bio-tek).

### *In vitro* release study

RAW264.7 were seeded into 12-well plates at a density of 2 × 10^4^ cells/ml. After 24 h, cells were pre-loaded with DiR-labeled NPs for 2 h, washed three times with ice-cold PBS, The NPs loaded macrophages were incubated with two different fresh release media, DMEM (no phenol red) and 10% FBS, DMEM (no phenol red) and 10% FBS with 500 ng/ml LPS and 200 ng/ml IFN-γ, respectively (*n* = 3). LPS and IFN-γ were added to mimic tumor inflammation microenvironment to activate RAW264.7 [42, 43]. The media was collected at various time intervals. The levels of fluorescence were measured on a Shimadzu RF5000 fluorescent spectrophotometer.

### Avascular glioma spheroids penetration of M-NPs

Three-dimensional spheroids of U87 cells were prepared by a lipid overlay method as reported previously [44]. Briefly, a 48-well plate was pretreated with 200 μl 2% (w/v) agarose gel to prevent cell adhesion, U87 cells were seeded into each well at the density of 2 × 10^3^ cells/well, then the plates were gently agitated for 5 min and cultured at 37°C in the presence of 5% CO_2_ for 7 days. Glioma spheroids were incubated with coumarin-6 labeled NPs and M-NPs respectively for 12 hours, with the final coumarin-6 concentration at 100 ng/ml in each well. After that, glioma spheroids were rinsed with PBS for three times, fixed with 4% paraformaldehyde, transferred to a chambered covered slip, and analyzed by confocal microscopy (LSM710, Leica, Germany).

### *In vivo* imaging of M-NPs in orthotopic U87 glioma mice

The orthotopic U87 glioma bearing mice model was established by slowly injecting U87 cells (5 × 10^5^ cells/5 μl in pH 7.4 PBS) into right corpus striata of nude mice with the help of a stereotaxic apparatus. Three weeks later, six nude mice bearing intracranial U87 glioma were divided into two groups randomly (*n* = 3), the mice in two groups were intravenously administrated with 200 μl DiR-NPs and M-NPs-DiR via the tail vein. The distribution of fluorescence was observed at predetermined time points (2, 4, 8, 12, 24 h) via an *in vivo* imaging system (IVIS Spectrum, Caliper, USA). Twenty-four hours after administration, the mice were sacrificed and the brains were harvested and imaged.

### Brain distribution of M-NPs in orthotopic U87 glioma mice

Coumarin-6-loaded NPs and M-NPs were injected to the orthotopic U87 glioma bearing mice respectively (*n* = 3) by tail vein. The mice were anesthetized 12 hours later, and their hearts were perfused with saline followed by 4% paraformaldehyde. The brains were collected, fixed with 4% paraformaldehyde overnight, and dehydrated using 15% glucose in PBS followed by 30% glucose in PBS. Then the tumors were embedded in Tissue Tek O.C.T. compound, frozen at −80°C and sectioned as slides at 5 μm thicknesses. The slides were subjected to confocal microscopy analysis after stained with DAPI for 10 min and rinsed with PBS.

### Statistical analysis

All the data were presented as mean ± standard deviation. Unpaired student's *t* test was used for between two-group comparisons. Statistical significance was defined as *p* < 0.05.
